# Supplementary figures and images for: Bean Consumption during Childhood Is Associated with Improved Nutritional Outcomes in the First Two Years of Life
Source: Nutrients. 2024 Apr 10;16(8):1120. doi: 10.3390/nu16081120 (PMC11053677; doi:10.3390/nu16081120)

## SUPPLEMENTARY MATERIALS

**Supplementary Figure S1. Flow chart of participants**

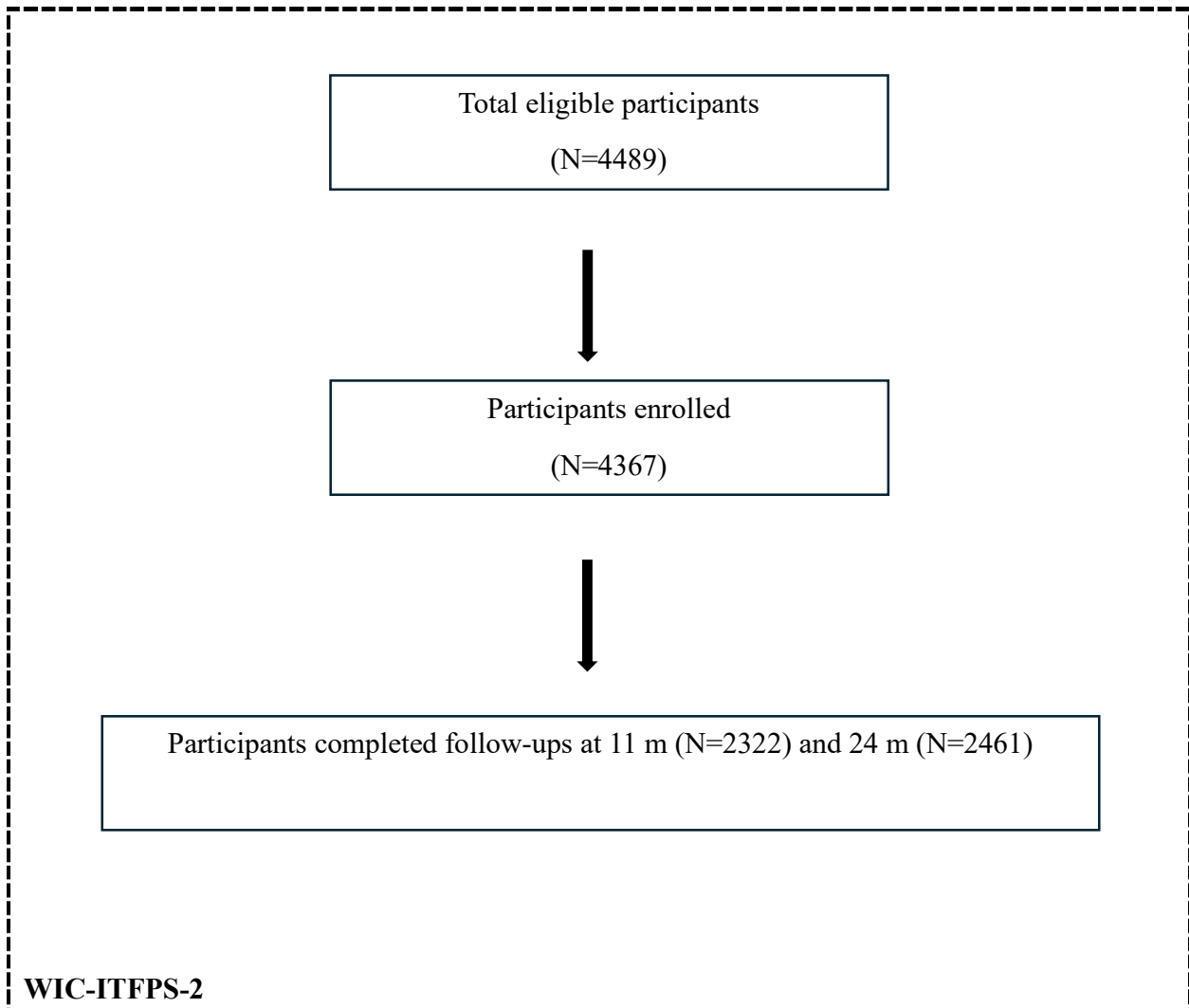

Supplement: Supplementary file 1 [file nutrients-16-01120-s001.zip › nutrients-2916991-supplementary.pdf]
